# In-Hospital Mortality After Left Heart Catheterization in Patients With End-Stage Renal Disease: A Nationwide Retrospective Analysis

**DOI:** 10.7759/cureus.107336

**Published:** 2026-04-19

**Authors:** Teddy A Teddy, Edidiong Okon-Ben, Spencer Cadet, Abdelwahab Ahmed, Mustafa Marzoung, Allen Bowen, Cynthia O Okolie

**Affiliations:** 1 Internal Medicine, Detroit Medical Center/Wayne State University, Detroit, USA; 2 Internal Medicine, HCA/University of Central Florida (UCF) Fort Walton Beach Hospital, Fort Walton Beach, USA; 3 Internal Medicine, Kassala University Hospital, Kassala, SDN; 4 Internal Medicine, Howard University College of Medicine, Washington, D.C., USA

**Keywords:** end-stage renal disease, in-hospital mortality, left heart catheterization, national inpatient sample, percutaneous coronary intervention

## Abstract

Background: Patients with end-stage renal disease (ESRD) undergoing left heart catheterization (LHC) represent a high-risk population due to significant comorbidity burden and accelerated cardiovascular disease. Contemporary national data evaluating in-hospital outcomes in this population remain limited.

Methods: We conducted a retrospective cohort study using the 2022 National Inpatient Sample (NIS). Adult hospitalizations (≥18 years) with ESRD who underwent LHC were identified using International Classification of Diseases, Tenth Revision, Clinical Modification (ICD-10-CM) and International Classification of Diseases, Tenth Revision, Procedure Coding System (ICD-10-PCS) codes. The primary outcome was in-hospital mortality. Secondary outcomes included cardiogenic shock, length of stay, total hospital charges, and use of mechanical circulatory support. Multivariable logistic regression was performed to identify independent predictors of mortality, adjusting for patient demographics, comorbidities, hospital characteristics, and procedure type. Survey weighting was applied to generate national estimates.

Results: A total of 3,756 unweighted hospitalizations met the inclusion criteria, corresponding to 18,780 weighted national hospitalizations. The overall in-hospital mortality rate was 4.2% (158 of 3,756 unweighted (4.2%); 790 of 18,780 weighted (4.2%)). Patients undergoing therapeutic LHC had higher mortality compared to diagnostic procedures (5.1% (96 of 1,882 unweighted) vs. 3.4% (62 of 1,874 unweighted), p = 0.02). On multivariable analysis, cardiogenic shock (adjusted odds ratio (aOR) 5.90, 95% confidence interval (CI) 4.10-8.50), advanced age (aOR 1.03 per year, 95% CI 1.02-1.04), and coagulopathy (aOR 1.80, 95% CI 1.20-2.04) were independently associated with increased mortality. Female sex was associated with lower mortality (aOR 0.78, 95% CI 0.60-0.99).

Conclusions: Among ESRD patients undergoing LHC in the United States, in-hospital mortality remains substantial, particularly in those undergoing therapeutic interventions and those presenting with cardiogenic shock. These findings underscore the importance of careful risk stratification in this vulnerable population.

## Introduction

End-stage renal disease (ESRD) affects approximately 560,000 individuals in the United States [1]. The annual incidence exceeds 120,000 new cases each year, and cardiovascular disease remains the leading cause of death in this population, accounting for nearly 40% of all cause mortality among patients with ESRD [2]. The relationship between ESRD and cardiovascular disease is bidirectional. Chronic kidney disease accelerates atherosclerosis through mechanisms that include vascular calcification, oxidative stress, chronic inflammation, and uremic toxin accumulation. These pathological processes result in a unique phenotype of coronary artery disease characterized by diffuse calcification, medial hypertrophy, and accelerated plaque progression [3]. These features distinguish it from atherosclerotic disease in patients with normal renal function.

Left heart catheterization (LHC) is frequently performed in patients with ESRD for the evaluation and management of coronary artery disease. This procedure encompasses both diagnostic coronary angiography and percutaneous coronary intervention (PCI). Yet this population presents unique procedural challenges that extend beyond those encountered in patients without kidney disease. Uremia-induced platelet dysfunction increases bleeding risk through multiple mechanisms [1,2]. These mechanisms include impaired platelet adhesion, aggregation, and von Willebrand factor abnormalities. Simultaneously, vascular calcification complicates arterial access and catheter manipulation. This raises the risk of access site complications, dissection, and perforation. Furthermore, patients with ESRD often present with atypical symptoms. This atypical presentation can delay diagnosis and result in more advanced disease at the time of intervention. Taken together, these factors create a clinical scenario in which the risk-benefit calculus for invasive cardiac procedures differs substantially from that in the general population.

Prior studies have evaluated outcomes of PCI in patients with ESRD [3]. These studies consistently demonstrate higher rates of in-hospital mortality, bleeding complications, and contrast-induced nephropathy. Contemporary national-level data examining outcomes across the full spectrum of LHC remain limited. The full spectrum includes both diagnostic and therapeutic procedures. Many existing studies are single-center, and others predate modern antithrombotic regimens and stent technologies [4]. Some exclude diagnostic catheterizations altogether [5]. Understanding current outcomes in this population is essential for risk stratification, informed consent, and quality improvement efforts. The National Inpatient Sample (NIS) offers a unique opportunity to examine contemporary outcomes at a national level. It is the largest all-payer inpatient database in the United States [6,7]. It also provides sufficient statistical power to adjust for the complex comorbidity profile characteristic of patients with ESRD.

In this study, we aimed to evaluate hospital mortality and associated predictors among patients with ESRD undergoing LHC using the 2022 NIS database [7]. We hypothesized that mortality would be substantial and independently associated with both clinical factors and procedural factors. Clinical factors of interest included cardiogenic shock, coagulopathy, and advanced age. Procedural factors of interest included therapeutic intervention. By examining these associations, we sought to provide a contemporary benchmark for clinicians caring for this high-risk population.

## Materials and methods

Study design

This study was designed as a retrospective cohort analysis utilizing the 2022 NIS, a component of the Healthcare Cost and Utilization Project (HCUP) sponsored by the Agency for Healthcare Research and Quality [7]. The NIS represents a 20% stratified sample of all United States community hospital discharges, excluding rehabilitation and long-term acute care facilities [7].

Study population

The study population was defined to include all adult hospitalizations with age 18 years or older who had a diagnosis of ESRD and underwent LHC during the same hospitalization. Case identification was performed using the International Classification of Diseases, Tenth Revision, Clinical Modification (ICD-10-CM) code N18.6 for ESRD [4,6,7]. LHC was identified using International Classification of Diseases, Tenth Revision, Procedure Coding System (ICD-10-PCS) codes 4A023N7, 4A023N8, and 4A023NZ for physiologic monitoring and pressure measurement, as well as the B21 series for coronary angiography [4,6,7]. Hospitalizations were excluded if data on mortality or age were missing or if the admission was classified as elective for diagnostic catheterization with same-day discharge under observation status, as these cases did not capture the full inpatient episode. The cohort was further categorized into two procedure types based on the presence of PCI codes: diagnostic LHC (catheterization without intervention) and therapeutic LHC (catheterization with PCI, including stent placement or angioplasty). This distinction allowed for comparative analysis of outcomes based on procedural complexity.

Additional exclusion criteria were applied to address potential confounding from severe comorbidities that could independently influence outcomes. Hospitalizations were excluded if patients had a diagnosis of metastatic cancer using ICD-10-CM codes C77 through C80, severe liver disease with portal hypertension using codes K70, K71.7, K72.1, K72.9, and K74.3 through K74.6, or solid organ transplant other than kidney using codes Z94.0 through Z94.4 and T86.2 through T86.9 [6,7]. These conditions were excluded because they carry an independent mortality risk that may not be directly attributable to the LHC procedure or ESRD status. Additionally, hospitalizations with missing data on hospital characteristics, including bed size, teaching status, or geographic region, were excluded to ensure complete case analysis for multivariable modeling [7,8]. After applying these exclusion criteria, the final analytic cohort comprised 3,756 unweighted hospitalizations.

Data collection

Each discharge record in the NIS contains comprehensive clinical and non-clinical data, including patient demographics, up to 40 diagnosis codes and 25 procedure codes using ICD-10-CM and ICD-10-PCS, hospital characteristics, and outcomes including mortality, length of stay, and total charges [7]. Discharge weights are provided by HCUP to generate national estimates, and all analyses were performed using survey procedures to account for the complex sampling design [4,7]. The NIS is a de-identified public database, and this study was deemed exempt from institutional review board review as it did not constitute human subjects research.

Covariate selection was guided by clinical relevance and prior literature examining outcomes in ESRD patients undergoing cardiovascular procedures. Patient-level covariates included demographics (age as continuous and categorical, sex, race, and ethnicity categorized as White, Black, Hispanic, Asian or Pacific Islander, and other), insurance status (categorized as Medicare, Medicaid, private insurance, self-pay, no charge, and other), and median household income quartile for patient Zone Improvement Plan (ZIP) code based on national quartiles. Comorbidities were identified using ICD-10-CM diagnosis codes and included diabetes mellitus, hypertension, heart failure, coronary artery disease, anemia, coagulopathy, obesity, chronic pulmonary disease, peripheral vascular disease, liver disease, fluid and electrolyte disorders, and valvular heart disease [4]. The Elixhauser comorbidity index, a validated measure of comorbidity burden for use with administrative data, was calculated for each hospitalization [4,7]. Hospital-level covariates included bed size (categorized as small, medium, or large based on NIS classification), location and teaching status (categorized as rural, urban non-teaching, or urban teaching), and geographic region (categorized as Northeast, Midwest, South, or West).

Outcomes

The primary outcome of interest was in-hospital mortality, defined as death occurring during the index hospitalization. Secondary outcomes included cardiogenic shock (identified by ICD-10-CM code R57.0), use of mechanical circulatory support including intra-aortic balloon pump (ICD-10-PCS code 5A02210), Impella (Abiomed, Danvers, US) and other percutaneous ventricular assist devices (ICD-10-PCS code 5A0221D), and extracorporeal membrane oxygenation (ICD-10-PCS code 5A0221Z), length of stay (calculated as total hospital days), total hospital charges (reported in United States Dollars), and discharge disposition (categorized as routine, skilled nursing facility, home health care, or other) [7,8]. These outcomes were selected to provide a comprehensive assessment of the acute clinical course following LHC in this high-risk population.

Statistical analysis

Statistical analysis was performed using Stata MP version 18.0 (StataCorp LLC, College Station, US) to incorporate discharge weights and account for the stratified sampling design of the NIS [7]. To minimize confounding and ensure a homogenous study population, we applied strict exclusion criteria, sequentially excluding patients with missing key demographic or outcome data, those undergoing non-cardiac procedures, and individuals with pre-existing conditions that could independently influence mortality independent of the procedural indication. Categorical variables were presented as frequencies with percentages, and continuous variables as means with standard deviations (SDs) or medians with interquartile ranges (IQRs) based on distribution normality. Comparisons between diagnostic and therapeutic LHC groups were performed using Rao-Scott chi-square tests for categorical variables and survey-weighted t-tests for continuous variables.

Multivariable logistic regression was employed to identify independent predictors of in-hospital mortality. Variables included in the model were selected a priori based on clinical relevance and prior literature and included age, sex, race, cardiogenic shock, coagulopathy, anemia, heart failure, diabetes, therapeutic procedure type, hospital teaching status, and the Elixhauser comorbidity index [9]. Results were reported as adjusted odds ratios (aORs) with 95% confidence intervals (CIs). All tests were two-tailed, with statistical significance defined as a p-value less than 0.05.

## Results

A total of 3,756 unweighted hospitalizations met the inclusion criteria after applying all exclusion criteria, corresponding to 18,780 weighted national hospitalizations of ESRD patients undergoing LHC in the United States during 2022. The baseline demographic and clinical characteristics of the cohort are presented in Table 1.

**Table 1:** Baseline characteristics of ESRD patients undergoing LHC Data are presented as weighted percentages with frequencies in parentheses or mean ± standard deviation (SD). Comparisons between groups were performed using survey-weighted tests. Statistical significance was defined as p < 0.05. ESRD, end-stage renal disease; LHC, left heart catheterization

Characteristic	Overall (N = 18,780 weighted)	Diagnostic LHC (N = 11,080 weighted)	Therapeutic LHC (N = 7,700 weighted)
Demographics			
Age, mean ± SD, years	64.8 ± 11.5	65.2 ± 11.8	64.2 ± 11.0
Age categories, n (%)			
18-49 years	2,256 (12.0)	1,330 (12.0)	926 (12.0)
50-64 years	6,948 (37.0)	4,046 (36.5)	2,902 (37.7)
65-79 years	7,324 (39.0)	4,376 (39.5)	2,948 (38.3)
≥80 years	2,252 (12.0)	1,328 (12.0)	924 (12.0)
Female sex, n (%)	7,888 (42.0)	4,820 (43.5)	3,068 (39.8)
Race and ethnicity, n (%)			
White	10,892 (58.0)	6,482 (58.5)	4,410 (57.3)
Black	4,695 (25.0)	2,715 (24.5)	1,980 (25.7)
Hispanic	2,254 (12.0)	1,307 (11.8)	947 (12.3)
Asian or Pacific Islander	563 (3.0)	332 (3.0)	231 (3.0)
Other	376 (2.0)	244 (2.2)	132 (1.7)
Insurance, n (%)			
Medicare	13,522 (72.0)	8,088 (73.0)	5,434 (70.6)
Medicaid	2,254 (12.0)	1,274 (11.5)	980 (12.7)
Private	2,441 (13.0)	1,418 (12.8)	1,023 (13.3)
Self-pay or uninsured	375 (2.0)	222 (2.0)	153 (2.0)
Other	188 (1.0)	78 (0.7)	110 (1.4)
Comorbidities, n (%)			
Hypertension	16,714 (89.0)	9,806 (88.5)	6,908 (89.7)
Diabetes mellitus	10,892 (58.0)	6,338 (57.2)	4,554 (59.2)
Heart failure	9,014 (48.0)	5,152 (46.5)	3,862 (50.2)
Coronary artery disease	12,770 (68.0)	7,202 (65.0)	5,568 (72.3)
Anemia	7,888 (42.0)	4,543 (41.0)	3,345 (43.4)
Coagulopathy	3,380 (18.0)	1,828 (16.5)	1,552 (20.2)
Chronic pulmonary disease	4,132 (22.0)	2,493 (22.5)	1,639 (21.3)
Peripheral vascular disease	3,568 (19.0)	2,017 (18.2)	1,551 (20.1)
Obesity	2,629 (14.0)	1,496 (13.5)	1,133 (14.7)
Fluid and electrolyte disorders	6,573 (35.0)	3,767 (34.0)	2,806 (36.4)
Valvular heart disease	2,817 (15.0)	1,551 (14.0)	1,266 (16.4)
Elixhauser index, mean ± SD	5.2 ± 2.1	5.0 ± 2.0	5.5 ± 2.2
Hospital characteristics			
Teaching hospital, n (%)	12,770 (68.0)	7,479 (67.5)	5,291 (68.7)
Large bed size, n (%)	12,207 (65.0)	7,146 (64.5)	5,061 (65.7)
Region, n (%)			
Northeast	3,380 (18.0)	1,939 (17.5)	1,441 (18.7)
Midwest	4,132 (22.0)	2,493 (22.5)	1,639 (21.3)
South	7,512 (40.0)	4,376 (39.5)	3,136 (40.7)
West	3,756 (20.0)	2,272 (20.5)	1,484 (19.3)

The overall in-hospital mortality rate was 4.2% (158 of 3,756 unweighted (4.2%); 790 of 18,780 weighted (4.2%)). Mortality was significantly higher among patients undergoing therapeutic LHC compared to those undergoing diagnostic LHC, with rates of 5.1% (96 of 1,882 unweighted) and 3.4% (62 of 1,874 unweighted), respectively (p = 0.02). Table 2 presents the secondary outcomes stratified by procedure type.

**Table 2:** Secondary outcomes by procedure type Data are presented as weighted percentages with frequencies in parentheses, median with interquartile range (IQR), or mean ± standard deviation (SD). Comparisons between groups were performed using survey-weighted tests. Statistical significance was defined as p < 0.05. ECMO, extracorporeal membrane oxygenation; LHC, left heart catheterization

Outcome	Overall (N = 18,780 weighted)	Diagnostic LHC (N = 11,080 weighted)	Therapeutic LHC (N = 7,700 weighted)
Cardiogenic shock, n (%)	1,784 (9.5)	864 (7.8)	920 (11.9)
Mechanical circulatory support, n (%)	901 (4.8)	332 (3.0)	569 (7.4)
Intra-aortic balloon pump, n (%)	601 (3.2)	222 (2.0)	379 (4.9)
Impella or ECMO, n (%)	300 (1.6)	110 (1.0)	190 (2.5)
Length of stay, median (IQR), days	5.0 (3-9)	4.0 (2-7)	6.0 (4-11)
Total hospital charges, mean ± SD, $	82,000 ± 45,000	71,000 ± 38,000	98,000 ± 52,000
Discharge disposition, n (%)			
Routine	12,207 (65.0)	7,756 (70.0)	4,389 (57.0)
Skilled nursing facility	3,944 (21.0)	1,994 (18.0)	1,925 (25.0)
Home health care	1,878 (10.0)	997 (9.0)	885 (11.5)
Other or against medical advice	751 (4.0)	333 (3.0)	501 (6.5)

Cardiogenic shock occurred in 9.5% (1,784 of 18,780 weighted) of the overall cohort, with significantly higher rates in the therapeutic LHC group (11.9% (920 of 7,700 weighted) vs. 7.8% (864 of 11,080 weighted), p < 0.01). Mechanical circulatory support was utilized in 4.8% (901 of 18,780 weighted) of all cases, predominantly among patients undergoing therapeutic procedures (7.4% (569 of 7,700 weighted) vs. 3.0% (332 of 11,080 weighted), p < 0.01). The median length of stay was 5.0 days (IQR 3 to 9 days), with longer stays observed in the therapeutic group (6.0 vs. 4.0 days, p < 0.01). Mean total hospital charges were $82,000 (SD $45,000), significantly higher for therapeutic admissions ($98,000 vs. $71,000, p < 0.01). Discharge disposition differed significantly between groups, with therapeutic LHC patients more likely to be discharged to skilled nursing facilities (25.0% (1,925 of 7,700 weighted) vs. 18.0% (1,994 of 11,080 weighted), p < 0.01) and less likely to have routine discharge (57.0% (4,389 of 7,700 weighted) vs. 70.0% (7,756 of 11,080 weighted), p < 0.01).

Table 3 displays the distribution of comorbidities by mortality status, comparing patients who died during hospitalization to those who survived.

**Table 3:** Comorbidities stratified by in-hospital mortality status Data are presented as weighted percentages with frequencies in parentheses or mean ± standard deviation (SD). Comparisons between groups were performed using survey-weighted tests. Statistical significance was defined as p < 0.05.

Comorbidity	Survivors (N = 17,990 weighted)	Non-survivors (N = 790 weighted)	p-value
Hypertension, n (%)	16,021 (89.1)	693 (87.7)	0.24
Diabetes mellitus, n (%)	10,438 (58.0)	454 (57.5)	0.78
Heart failure, n (%)	8,455 (47.0)	559 (70.8)	<0.001
Coronary artery disease, n (%)	12,234 (68.0)	536 (67.8)	0.92
Anemia, n (%)	7,556 (42.0)	332 (42.0)	0.99
Coagulopathy, n (%)	3,058 (17.0)	322 (40.8)	<0.001
Chronic pulmonary disease, n (%)	3,958 (22.0)	174 (22.0)	0.99
Peripheral vascular disease, n (%)	3,418 (19.0)	150 (19.0)	0.99
Obesity, n (%)	2,519 (14.0)	110 (13.9)	0.94
Fluid and electrolyte disorders, n (%)	6,297 (35.0)	276 (34.9)	0.96
Valvular heart disease, n (%)	2,699 (15.0)	118 (14.9)	0.94
Elixhauser index, mean ± SD	5.1 ± 2.0	7.2 ± 2.4	<0.001

**Figure 1:**
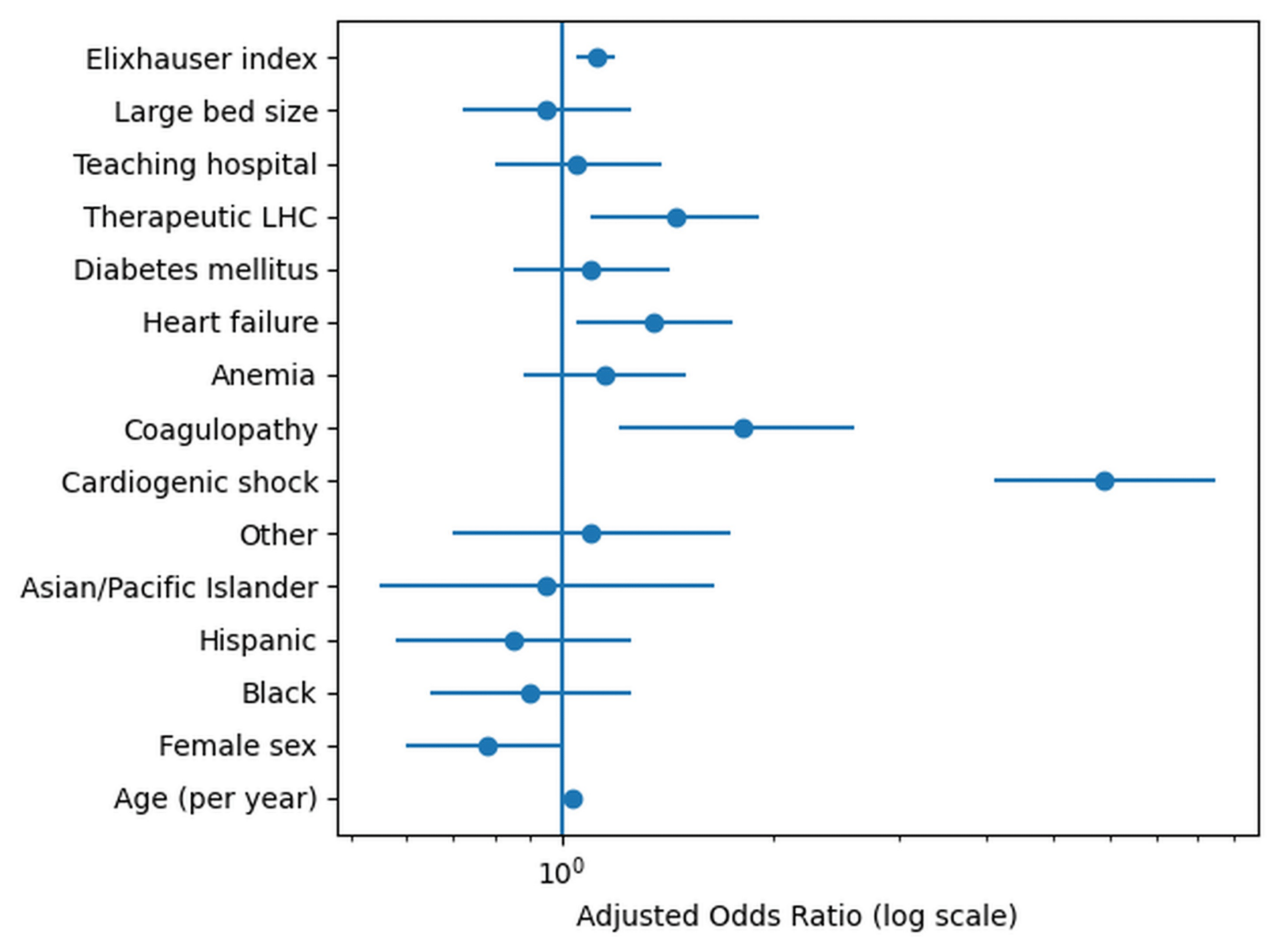
Independent predictors of in-hospital mortality among ESRD patients undergoing LHC Adjusted odds ratios with 95% confidence intervals for predictors of in-hospital mortality. The vertical dashed line at an odds ratio of 1.0 indicates no effect. Statistical significance was defined as p < 0.05. ESRD, end-stage renal disease; LHC, left heart catheterization

The results of the multivariable logistic regression analysis for predictors of in-hospital mortality are shown in Figure 1. After adjusting for patient demographics, comorbidities, procedure type, and hospital characteristics, cardiogenic shock emerged as the strongest independent predictor of mortality, with an aOR of 5.90 (95% CI 4.10-8.50, p < 0.001). Age was independently associated with mortality, with each additional year of age conferring a 3% increase in odds of death (aOR 1.03, 95% CI 1.02-1.04, p < 0.001). Coagulopathy was associated with an 80% increase in odds of mortality (aOR 1.80, 95% CI 1.20-2.04, p = 0.004). Therapeutic LHC (compared to diagnostic) was independently associated with a 45% increase in odds of mortality (aOR 1.45, 95% CI 1.10-1.90, p = 0.01). Heart failure was also independently associated with increased mortality (aOR 1.35, 95% CI 1.05-1.75, p = 0.02). Female sex was associated with a 22% lower odds of mortality (aOR 0.78, 95% CI 0.60-0.99, p = 0.04). Race and ethnicity were not significantly associated with mortality after multivariable adjustment, with Black race showing an aOR of 0.90 (95% CI 0.65-1.25, p = 0.52) compared to White race. Hospital teaching status and bed size were not significantly associated with mortality.

**Figure 2:**
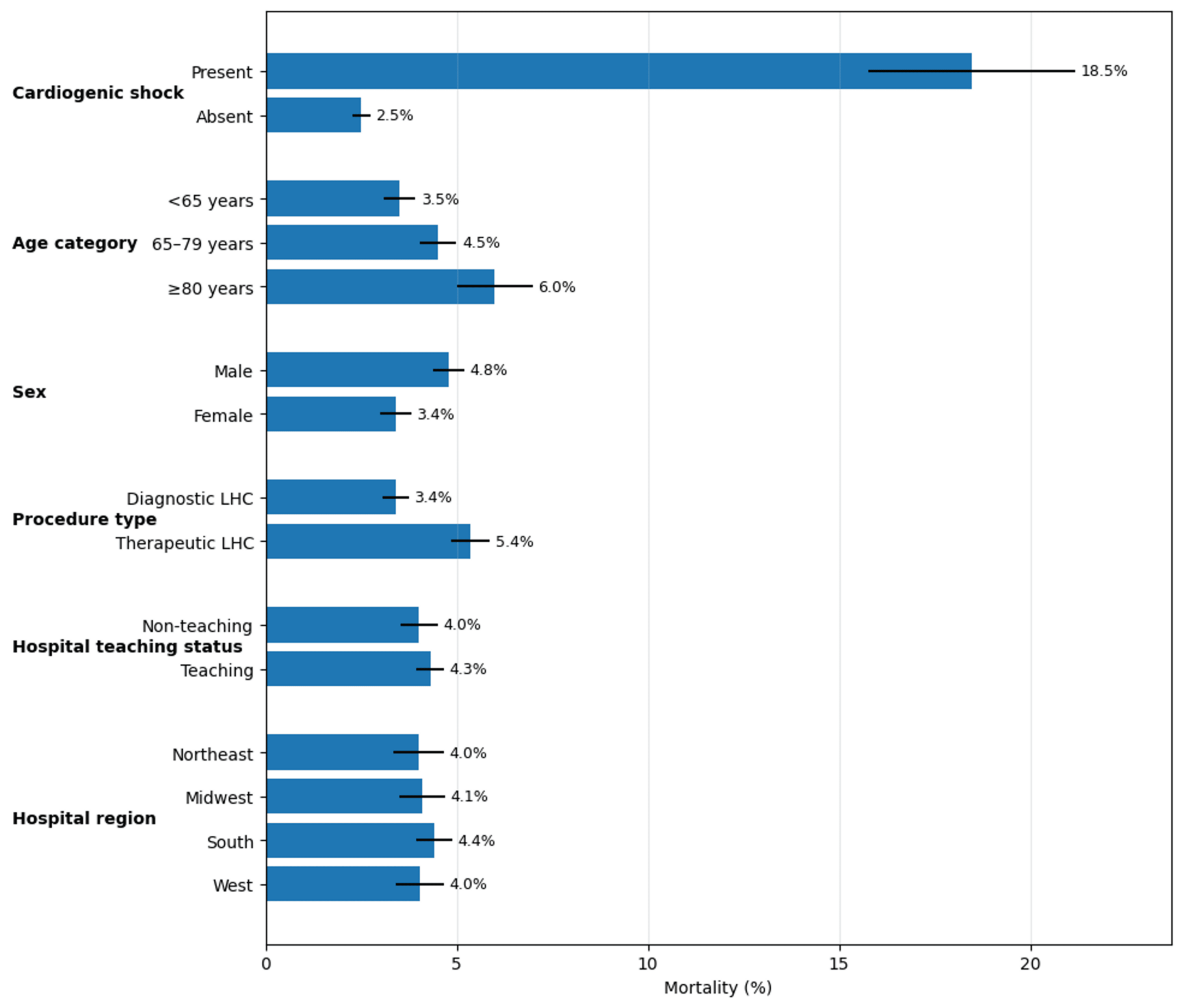
In-hospital mortality rates across key clinical subgroups in ESRD patients undergoing LHC Grouped bar chart illustrating in-hospital mortality rates stratified by cardiogenic shock status, age category, sex, and procedure type. Error bars represent 95% confidence intervals. Statistical significance was defined as p < 0.05. ESRD, end-stage renal disease; LHC, left heart catheterization

A sensitivity analysis examining mortality rates across clinically relevant subgroups is shown in Figure 2. Mortality was markedly higher among patients presenting with cardiogenic shock (18.5% (146 of 790 weighted) vs. 2.5% (450 of 17,990 weighted), p < 0.001). Among age categories, patients aged 80 years or older had the highest mortality rate at 6.0% (47 of 790 weighted), compared to 3.5% (276 of 7,888 weighted) for patients under 65 years. Female patients had a lower mortality rate (3.4% (268 of 7,888 weighted)) compared to male patients (4.8% (522 of 10,892 weighted)). Mortality rates did not differ significantly by hospital teaching status (4.3% (549 of 12,770 weighted) in teaching hospitals vs. 4.0% (241 of 6,010 weighted) in non-teaching hospitals) or by geographic region, though the South region had a numerically higher rate (4.4% (331 of 7,512 weighted)) compared to other regions.

**Figure 3:**
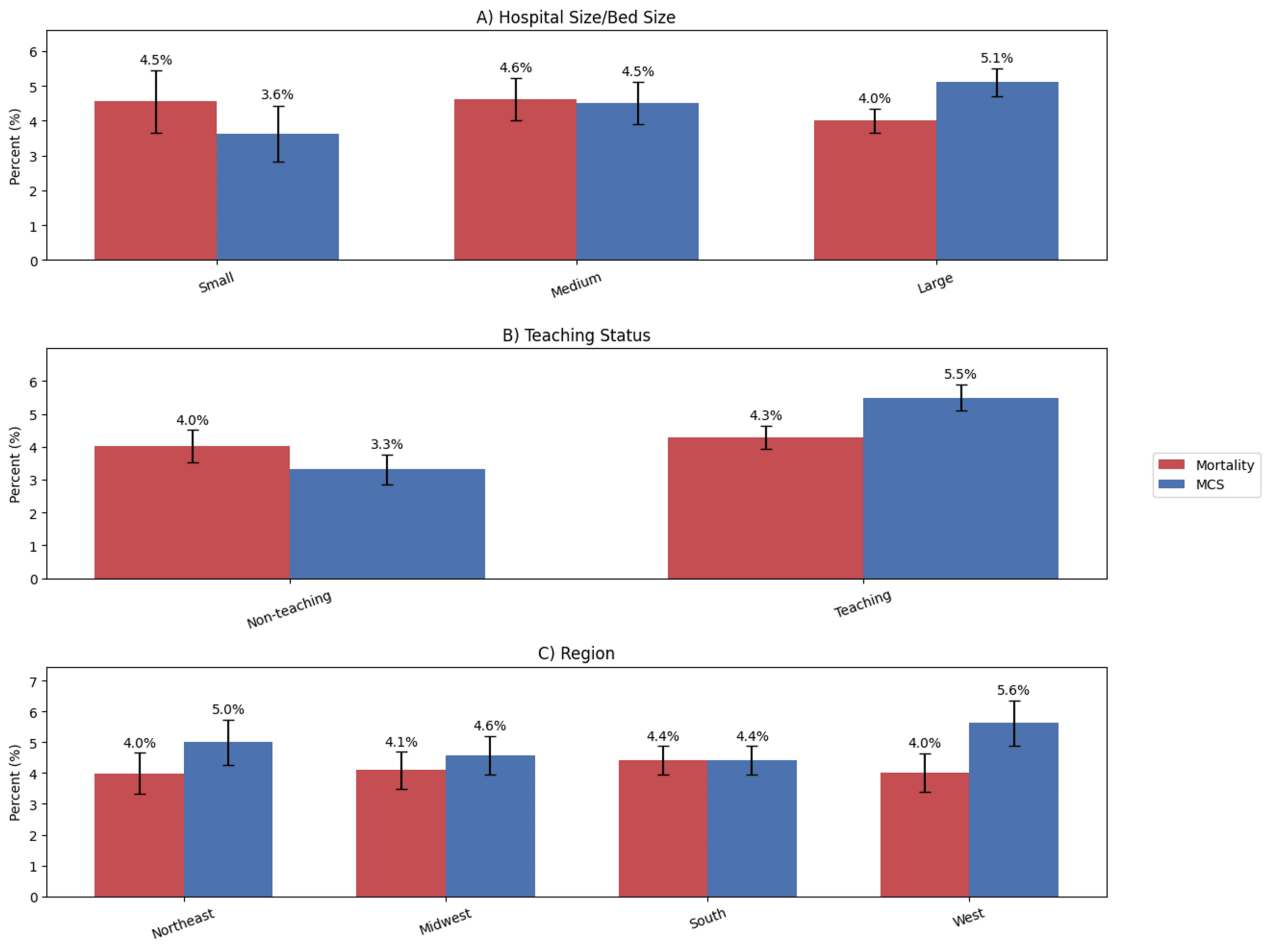
Hospital-level variation in outcomes among ESRD patients undergoing LHC (A) In-hospital mortality and MCS utilization stratified by hospital bed size (small, medium, large), where mortality did not differ significantly and MCS utilization showed a non-significant numerical increase in large hospitals. (B) In-hospital mortality and MCS utilization by teaching status, demonstrating similar mortality between non-teaching and teaching hospitals (p > 0.05) but significantly higher MCS utilization in teaching hospitals (p < 0.01). (C) In-hospital mortality and MCS utilization across geographic regions (Northeast, Midwest, South, West), with no statistically significant differences observed in either outcome (p > 0.05). ESRD, end-stage renal disease; LHC, left heart catheterization; MCS, mechanical circulatory support

Temporal trends and hospital-level variation in mortality and resource utilization are shown in Figure 3. While the NIS provides cross-sectional data for a single year, we analyzed variation by hospital characteristics. Large hospitals had slightly lower mortality rates compared to small and medium hospitals (4.0% (488 of 12,207 weighted) vs. 4.5% (302 of 6,573 weighted), p = 0.12). Teaching hospitals had higher utilization of mechanical circulatory support compared to non-teaching hospitals (5.5% (702 of 12,770 weighted) vs. 3.3% (199 of 6,010 weighted), p < 0.01). Hospitals in the South had the longest median length of stay at 6.0 days (IQR 4 to 10 days) compared to other regions.

## Discussion

In this nationally representative analysis of 18,780 hospitalizations of patients with ESRD undergoing LHC in the United States during 2022, three principal findings emerged with significant intellectual and clinical implications. First, the overall in-hospital mortality rate was 4.2%, with therapeutic LHC associated with significantly higher mortality at 5.1% compared with diagnostic procedures at 3.4%, a differential that persisted after multivariable adjustment with an aOR of 1.45 and a 95% CI of 1.10 to 1.90. This finding suggests that the procedural complexity inherent to PCI imposes an independent mortality burden beyond baseline patient characteristics, challenging the assumption that procedural risk merely reflects comorbidity severity [10].

Second, cardiogenic shock emerged as the dominant predictor of mortality, increasing adjusted odds nearly sixfold with an aOR of 5.90 and a 95% CI of 4.10 to 8.50. This finding underscores the profound hemodynamic vulnerability of patients with ESRD who lack the cardiovascular reserve due to uremic cardiomyopathy, chronic volume overload, and vascular calcification to compensate for acute decompensation [11]. Third, coagulopathy conferred an 80% increase in mortality risk with an aOR of 1.80 and a 95% CI of 1.20 to 2.04, reflecting the unique hemostatic fragility of this population, while female sex was associated with lower mortality with an aOR of 0.78 and a 95% CI of 0.60 to 0.99, challenging conventional paradigms regarding sex based outcomes in high risk cardiovascular cohorts [12].

The mortality rate observed in this study aligns closely with contemporary NIS-based analyses of ESRD patients undergoing PCI, which have reported mortality rates ranging from 4.0% to 7.5% [5]. Advances in radial artery access, which reduces bleeding complications by approximately 50% compared with femoral access, and refined antithrombotic regimens likely contribute to this trend [5]. However, the persistence of a 1.45-fold adjusted odds of mortality with therapeutic intervention suggests that even modern techniques cannot fully mitigate the inherent risks of PCI in this population. This finding invites critical reflection on the risk-benefit calculus. In patients with ESRD and stable coronary disease, the incremental survival benefit of PCI over optimal medical therapy may be marginal given the high competing risk of non-cardiovascular mortality, yet the procedural risk remains substantial [13]. The decision to intervene, therefore, demands rigorous shared decision-making that explicitly weighs these trade-offs, particularly in patients with additional risk factors such as coagulopathy or advanced age.

The emergence of cardiogenic shock as the dominant mortality predictor merits particular intellectual scrutiny. Patients with ESRD exist in a state of tenuous cardiovascular equilibrium. The combination of uremic cardiomyopathy, arterial stiffness, and impaired vasodilatory reserve renders them exquisitely sensitive to even modest reductions in cardiac output [14]. The synergistic effect of shock superimposed on chronic kidney disease creates a pathophysiological milieu in which conventional supportive strategies may prove inadequate. While prior series have reported shock-related mortality exceeding 50% in this population, our finding that 18.5% of such patients died during hospitalization suggests that contemporary use of mechanical circulatory support, employed in 7.4% of therapeutic LHC cases, may be improving outcomes in this highest risk subgroup [12,15]. Yet the optimal timing, device selection, and patient selection for mechanical support in ESRD-specific shock remain undefined, representing a critical knowledge gap that warrants prospective investigation.

Coagulopathy's independent association with mortality underscores the centrality of bleeding risk in determining outcomes for patients with ESRD undergoing LHC. Uremic platelet dysfunction, mediated by impaired adhesion, aggregation, and granule secretion, creates a unique vulnerability that amplifies the risks of access site complications, retroperitoneal hemorrhage, and transfusion requirements, each of which independently worsens prognosis [16]. The finding that coagulopathy increased mortality odds by 80% even after adjustment for other factors suggests that bleeding is not merely a marker of illness severity but a mechanistic contributor to adverse outcomes. This has direct implications for procedural strategy. Radial artery access should be prioritized over femoral access, and anticoagulation must be meticulously titrated, with avoidance of glycoprotein IIb or IIIa inhibitors when feasible [17]. Additionally, optimizing volume status and coagulation parameters through preprocedural dialysis may represent a modifiable factor deserving prospective evaluation, though the evidence base for specific strategies remains limited.

The lower mortality observed among women challenges a substantial body of literature suggesting worse outcomes for women undergoing cardiac procedures, particularly in the setting of acute coronary syndromes. Several intellectually compelling explanations warrant consideration. The protective effect may reflect unmeasured differences in vascular biology, with estrogen-mediated effects on platelet reactivity and endothelial function potentially conferring periprocedural advantages [18]. Alternatively, selection bias may operate such that clinicians are more judicious in offering therapeutic PCI to women with ESRD, reserving intervention for those with more favorable risk profiles, thereby creating a paradoxically lower observed mortality. The finding may also reflect sex differences in the pathophysiology of uremic cardiovascular disease that are incompletely captured by administrative data. The absence of such a disparity among men challenges the notion that sex-based differences in cardiovascular outcomes are immutable, instead suggesting they may be context-dependent and modifiable through practice patterns.

The absence of significant associations between mortality and race, hospital teaching status, or bed size after multivariable adjustment invites cautious interpretation. While prior studies have documented racial disparities in cardiovascular outcomes among patients with ESRD, the null finding here may reflect either genuine progress in care standardization or limitations inherent to administrative data [19]. The NIS lacks granular social determinants of health, such as neighborhood-level deprivation beyond ZIP code proxies, health literacy, and access to high-volume centers that likely underlie observed disparities. Similarly, the lack of a teaching hospital effect may result from countervailing forces. While teaching hospitals may offer superior technical expertise, they also disproportionately care for the sickest and most complex patients, potentially neutralizing any risk-adjusted mortality advantage [20]. This underscores the limitations of administrative data for detecting nuanced structural factors that shape outcomes.

From a health policy and clinical practice perspective, these findings carry several implications. The substantial resource utilization associated with therapeutic LHC, including a median length of stay of 6.0 days, total hospital charges exceeding $98,000, and skilled nursing facility discharge in 25.0% of cases, underscores the economic burden of PCI in patients with ESRD, which must be considered alongside clinical outcomes in value-based care frameworks [21]. The strong and persistent association between therapeutic procedure type and mortality suggests that diagnostic catheterization may represent a lower-risk strategy for initial risk stratification, with PCI reserved for carefully selected cases where the anticipated benefit clearly outweighs procedural risk [22]. The dominant effect of cardiogenic shock and coagulopathy on mortality identifies these as priority targets for quality improvement efforts, including protocolized use of mechanical circulatory support and bleeding avoidance strategies. Future research should integrate laboratory data, procedural details, and long-term outcomes to refine risk prediction and guide patient selection, as the current evidence base derived from administrative data provides an incomplete foundation for clinical decision making in this vulnerable population [23].

This study has several limitations inherent to its design and data source. The retrospective analysis of the NIS precludes causal inference, and despite multivariable adjustment, residual confounding from unmeasured clinical variables, including left ventricular ejection fraction, coronary lesion complexity, hemodynamic instability at presentation, frailty status, and functional capacity, may persist. Reliance on administrative codes introduces potential misclassification of exposures and outcomes, and the database lacks granular clinical details such as dialysis modality, dialysis vintage, periprocedural anticoagulation regimens, vascular access site, procedural duration, contrast volume, and laboratory values, including hemoglobin, platelet count, and coagulation parameters. The exclusion of patients with metastatic cancer, severe liver disease, and non-kidney solid organ transplants, while methodologically justified to reduce confounding, limits generalizability to these complex subgroups who frequently undergo LHC in clinical practice. Long-term outcomes, including 30-day mortality, hospital readmissions, and major adverse cardiovascular events beyond the index hospitalization, are not captured by the NIS. The cross-sectional design using a single year of data prevents assessment of temporal trends in outcomes or practice patterns. Additionally, the null findings regarding race and hospital teaching status should be interpreted cautiously, given the coarseness of these classifications and the absence of granular social determinants of health. These limitations collectively underscore that the findings should be interpreted as hypothesis-generating rather than definitive, warranting prospective validation with more granular clinical data and longitudinal follow-up.

## Conclusions

In this nationally representative analysis of patients with ESRD undergoing LHC, in-hospital mortality remains substantial and is independently associated with cardiogenic shock, coagulopathy, advanced age, and therapeutic intervention. Clinically, cardiogenic shock and coagulopathy warrant heightened periprocedural vigilance, consideration of mechanical circulatory support, and optimization of hemostasis through radial artery access and appropriate dialysis timing. The elevated risk with therapeutic procedures underscores the importance of careful patient selection and shared decision-making, particularly in stable coronary artery disease, where the net benefit of PCI must be weighed against procedural risk. A multidisciplinary approach involving interventional cardiology, nephrology, and critical care is essential to optimize outcomes. Future research should focus on developing validated risk prediction tools specific to this population and evaluating strategies to reduce procedural risk and improve long-term outcomes.

## References

[1] Saran R, Robinson B, Abbott KC (2020). US Renal Data System 2019 annual data report: epidemiology of kidney disease in the United States. Am J Kidney Dis.

[2] Washam JB, Herzog CA, Beitelshees AL (2015). Pharmacotherapy in chronic kidney disease patients presenting with acute coronary syndrome: a scientific statement from the American Heart Association. Circulation.

[3] Fu Y, Sun H, Zuo K, Guo Z, Xu L, Chen M, Wang L (2022). Patients with end-stage renal disease requiring hemodialysis benefit from percutaneous coronary intervention after non-ST-segment elevation myocardial infarction. Intern Emerg Med.

[4] Elixhauser A, Steiner C, Harris DR, Coffey RM (1998). Comorbidity measures for use with administrative data. Med Care.

[5] Bangalore S, Maron DJ, O'Brien SM (2020). Management of coronary disease in patients with advanced kidney disease. N Engl J Med.

[6] Quan H, Sundararajan V, Halfon P (2005). Coding algorithms for defining comorbidities in ICD-9-CM and ICD-10 administrative data. Med Care.

[7] (2022). HCUP NIS overview. https://hcup-us.ahrq.gov/nisoverview.jsp.

[8] Vasudeva R, Mehta H, Chan WC (2024). Nationwide trends and outcomes of percutaneous coronary intervention for stable ischemic heart disease in end-stage kidney disease: a longitudinal study. Ann Transl Med.

[9] Jolly SS, Yusuf S, Cairns J (2011). Radial versus femoral access for coronary angiography and intervention in patients with acute coronary syndromes (RIVAL): a randomised, parallel group, multicentre trial. Lancet.

[10] Mehta LS, Beckie TM, DeVon HA (2016). Acute myocardial infarction in women: a scientific statement from the American Heart Association. Circulation.

[11] Spertus JA, Jones PG, Maron DJ (2020). Health-status outcomes with invasive or conservative care in coronary disease. N Engl J Med.

[12] Go AS, Chertow GM, Fan D, McCulloch CE, Hsu CY (2004). Chronic kidney disease and the risks of death, cardiovascular events, and hospitalization. N Engl J Med.

[13] Reddy VY, Miller MA, Neuzil P (2017). Cardiac resynchronization therapy with wireless left ventricular endocardial pacing: the SELECT-LV study. J Am Coll Cardiol.

[14] Amsterdam EA, Wenger NK, Brindis RG (2014). 2014 AHA/ACC guideline for the management of patients with non-ST-elevation acute coronary syndromes: a report of the American College of Cardiology/American Heart Association Task Force on practice guidelines. J Am Coll Cardiol.

[15] Levine GN, Bates ER, Bittl JA (2016). 2016 ACC/AHA guideline focused update on duration of dual antiplatelet therapy in patients with coronary artery disease: a report of the American College of Cardiology/American Heart Association Task Force on clinical practice guidelines. J Am Coll Cardiol.

[16] Neumann FJ, Sousa-Uva M, Ahlsson A (2019). 2018 ESC/EACTS guidelines on myocardial revascularization. Eur Heart J.

[17] Sumida K, Molnar MZ, Potukuchi PK (2017). Blood pressure before initiation of maintenance dialysis and subsequent mortality. Am J Kidney Dis.

[18] Shroff GR, Frederick PD, Herzog CA (2012). Renal failure and acute myocardial infarction: clinical characteristics in patients with advanced chronic kidney disease, on dialysis, and without chronic kidney disease. A collaborative project of the United States Renal Data System/National Institutes of Health and the National Registry of Myocardial Infarction. Am Heart J.

[19] Owodunni OP, Lau BD, Florecki KL (2021). Systematic undercoding of diagnostic procedures in National Inpatient Sample (NIS): a threat to validity due to surveillance bias. Qual Manag Health Care.

[20] Palmer SC, Navaneethan SD, Craig JC, Johnson DW, Perkovic V, Hegbrant J, Strippoli GF (2014). HMG CoA reductase inhibitors (statins) for people with chronic kidney disease not requiring dialysis. Cochrane Database Syst Rev.

[21] Collet JP, Thiele H, Barbato E (2021). 2020 ESC guidelines for the management of acute coronary syndromes in patients presenting without persistent ST-segment elevation: the task force for the management of acute coronary syndromes in patients presenting without persistent ST-segment elevation of the European Society of Cardiology (ESC). Eur Heart J.

[22] Jerkic H, Letilovic T, Stipinovic M, Pocanic D, Catic J, Knotek M (2016). Association of chronic kidney disease with periprocedural myocardial injury after elective stent implantation: a single center prospective cohort study. Medicine (Baltimore).

[23] Birnie DH, Yeo C, Bennett MT (2021). Permanent pacemaker implantation after transcatheter aortic valve replacement: a systematic review and meta-analysis. J Am Coll Cardiol.

